# Synthesis, Characterization, Antimicrobial Activity, Docking, and In Silico ADMET Analysis of Some New 2,3‐Dihydrobenzo[b][1,4]thiazepine Derivatives

**DOI:** 10.1155/bmri/5076755

**Published:** 2026-05-30

**Authors:** Felix Odame, Reuben Ayivor-Djanie, Tatenda Madanhire, Jaclyn Mann, David Neglo, Salifu Nanga, Emmanuel Hamenoo, Cedric Amengor, Susana Acheampong, Prince Biniyam, Takalani Mulaudzi

**Affiliations:** ^1^ Department of Basic Sciences, University of Health and Allied Sciences, Ho, Ghana, uhas.edu.gh; ^2^ Department of Biomedical Sciences, University of Health and Allied Sciences, Ho, Ghana, uhas.edu.gh; ^3^ Department of Chemistry, University of South Africa, Johannesburg, Gauteng, South Africa, unisa.ac.za; ^4^ HIV Pathogenesis Programme, Nelson R. Mandela School of Medicine, University of KwaZulu-Natal, Durban, South Africa, ukzn.ac.za; ^5^ Department of Pharmaceutical Chemistry, School of Pharmacy, University of Health and Allied Sciences, Ho, Ghana, uhas.edu.gh; ^6^ University of Witwatersrand, School of Anatomical Sciences, Johannesburg, South Africa, wits.ac.za

**Keywords:** antimicrobial activity, benzothiazepine, in silico ADMET analysis, molecular docking

## Abstract

A series of 2,3‐dihydrobenzo[b][1,4]thiazepine derivatives have been synthesized and characterized using IR, NMR, GC‐MS and microanalysis. The compounds were found to exhibit variable activity against *Escherichia coli*, *Klebsiella pneumoniae*, *Enterococcus faecalis* and *Salmonella typhi* with MIC values ranging from 1.88–15 mg/mL. The best activity was observed in compounds 4 and 6 with MIC of 1.875 mg/mL against *E*. *faecalis.* Compound 6 was also the most active against *S*. *aureus* with MIC of 1.875 mg/mL. The in silico computational docking revealed that the compounds have excellent binding to both multiple targets of *E. coli* and *Staphylococcus aureus* dihydrofolate reductase enzymes (PDB ID: 6XG5 and 6P9Z) and good binding to the *S. aureus* dihydropteroate synthase enzyme (PDB ID: 1ad4). The computational pharmacokinetic study showed that the highly orally bioavailable compounds were not P‐gp (p‐glycoprotein substrate). The compounds were found to be excellent hERG blockers.

## 1. Introduction

A 1,5‐benzothiazepine derivatives have been found to exhibit specific mitochondrial activity [1]. This activity has been observed to be linked to the nature of the substituents in the 1,5‐benzothiazepine ring and the conjugation of the fused heterocyclic ring at the nitrogen site [2]. The *N*‐nitroso derivatives of some 1,5‐benzothiazepine derivatives have been found to have cancer‐inhibiting properties [3]. Benzothiazepine containing compounds have been reported to exhibit anti‐HIV [4], antihypertensive [5], and antibacterial activity [6]. Diltiazem is a benzothiazepine derivative, which is a calcium channel blocker [7], an angiotensin‐converting enzyme inhibitor [8], and an anticonvulsant and tranquilizing agent [9] and has other medicinal uses [10]. Benzothiazepine derivatives inhibit bile acid transport that regulates bile acid reabsorption [11–13], thus increasing bile acid flow to the colon, thereby promoting the intestinal tract to secrete more water and facilitate defecation, so as to naturally improve natural defecation in patients.

Some 1,3,4‐oxadiazolyl‐benzodiazepines and benzothiazepines have been synthesized and examined for biological activity. The bioactivity of the compounds was found to be greatly improved by the presence of thiophene, pyridine, and furan on the benzothiazepine and benzodiazepine rings. The compounds showed good antibacterial and antifungal activity [14]. Some 1,5‐benzothiazepines have been accessed through the reaction of substituted chalcones with 2‐aminothiophenol using conventional method and with microwave irradiation. The compounds were found to be active against a melanoma cell line, used as a model for human breast cancer cell lines (MDA‐MB‐435) [15]. An efficient one‐pot, multicomponent synthesis of 2,3‐dihydro‐1,5‐benzothiazepines has been achieved by aluminum nitrate catalysis. The products were obtained in good yields in shorter reaction times [16]. Antimicrobial resistance has become a common challenge to the treatment of infectious diseases, hence posing a global threat. Organisms implicated in antimicrobial resistance include methicillin‐resistant *Staphylococcus aureus* (MRSA), vancomycin‐resistant *Enterococcus* (VRE), multidrug‐resistant *Mycobacterium tuberculosis* (MDR‐TB), and carbapenemase‐producing Enterobacterales (CPE) [17]. It is estimated that bacterial antimicrobial resistance was directly responsible for 1.27 million global deaths in 2019 and contributed to 4.95 million deaths [18, 19]. Current antimicrobials such as penicillins, cephalosporins, and quinolones that are used in the treatment of these infections are losing their efficacy due to antimicrobial resistance, hence the need to search for new antimicrobial agents to augment the fight against antimicrobial resistance [20].

Some synthesized thiazepine and diazepine derivatives have been reported to possess good antitubercular and antimicrobial activity when tested against some bacteria (*S. aureus*, *Staphylococcus pyogenes*, *Escherichia coli*, and *Pseudomonas aeruginosa*), fungi (*Candida albicans*, *Aspergillus niger*, and *Aspergillus clavatus*), and protozoa (*Entamoeba histolytica*, *Giardia lamblia*, *Trypanosoma cruzi*, and *Leishmania mexicana*) [14]. Some clinically administered drugs have been reported to contain the 1,5‐benzothiazepine moiety, including diltiazem, clentiazem, thiazesim, quetiapine, and clothiapine. The 1,5‐benzothiazepine nucleus was found to be active against a wide range of disease‐causing organisms [21]. The facile synthesis of some 1,4‐benzothiazepine derivatives has been achieved by the acid‐catalyzed hydrolysis of chalcones and 2‐aminothiophenol. The compounds were found to be good potential antibacterial agents [22]. A series of 1,5‐benzothiazepine derivatives have been reported to exhibit significant anti‐inflammatory activity in models of acute inflammation, where the compounds showed considerable activity compared with diclofenac as a standard [23]. A series of novel 4‐[substituted‐2‐hydroxy‐phenyl]‐2‐(4 ^′^‐dimethylamino‐phenyl)‐2,3‐dihydro‐1,5‐benzothiazepines have been synthesized. The compounds were not active against *E. coli* but showed moderate activity against *S. aureus*, significant activity against *B. subtilis*, and antifungal activity [24]. A variety of 1,5‐benzothiazepines have been prepared by the cyclo‐condensation reaction of *o*‐aminothiophenol derivatives with carbonyl and other functionalities to give benzothiazepine derivatives, which are reported to have remarkable biological activity [25]. The synthesis of some [3, 1]‐benzothiazepines and [3, 1]‐benzoxazepines by the reaction of C‐allylanilines and isothiocyanates or isocyanates has been reported. The compounds exhibited good antitumor activity for HL‐60 cells [26].

We herein report the synthesis, characterization, antimicrobial, in silico molecular docking, and in silico absorption, distribution, metabolism, excretion, and toxicity (ADMET) analysis of some new benzothiazepine derivatives.

## 2. Materials and Method

### 2.1. Chemistry

The chemicals to be used in the synthesis, 2‐aminothiophenol, aldehydes: 4‐methylbenzaldehyde, 4‐chlorobenzaldehyde, 2‐chlorobenzaldehyde, 3‐methylbenzaldehyde, 3‐nitrobenzaldehyde, benzaldehyde, salicylaldehyde, N, N‐diethylaminobenzaldehyde, and 4‐methylpent‐3‐en‐2‐one were purchased from Sigma‐Aldrich (United States). With the Merck Chemicals (South Africa) providing the ethanol, acetone and methanol. The compounds were utilized just as supplied, requiring no additional purification. Ethyl acetate: petroleum ether (1:1) was used to monitor the reaction using aluminum‐coated TLC (1 × 4 cm). Using tetramethylsilane as an internal standard and deuterated dimethyl sulfoxide as a solvent, ^1^H (400 MHz) and ^13^C (100 MHz) NMR spectra were acquired on a Bruker Avance AV 400 MHz spectrometer. The unit of measurement for chemical shifts is ppm. A Bruker Platinum ATR Spectrophotometer Tensor 27 was used to record the FT‐IR spectra. A Vario Elementar Microcube ELIII was used for microanalysis. The melting points were determined by means of a Stuart Lasec SMP30 and displayed uncorrected, whereas the masses were measured using a PerkinElmer GC Clarus 580 Gas Chromatograph interfaced to a PerkinElmer (Clarus SQ 8 S) Mass Spectrometer with a ZB‐5HTMS (5% diphenyl/95% dimethyl polysiloxane) fused capillary column (30 × 0.25 *μ*mID × 0.25 *μ*m DF). The oven was set to start at 100°C (isothermal for 2 min), rise by 10°C per minute to 200°C, then rise by 5°C per minute to 280°C, where it was maintained for 6 min. The electron ionization system was run in electron impact mode with an ionization energy of 70 eV for GC‐MS detection. Helium gas (99.9999%) was utilized as a carrier gas with the volume of the injection of 1 *μ*L and a steady flow rate of 1 mL/min. The ion‐source temperature was 220°C, whereas the injector temperature was kept at 250°C. Mass spectra were obtained at 70 eV, with pieces ranging from 50 to 500 Da with a scan interval of 1 s. The GC/MS ran for 50 min total, with a solvent delay of 0 to 3 min. Turbo‐Mass was the mass detector utilized in this investigation, and Turbo‐Mass Ver‐6.1.0 was the software employed to process mass spectra and chromatograms. The National Institute of Standards and Technology′s (NIST) database, which contains over 62,000 patterns, was employed for mass‐spectrum GC‐MS interpretation [27–29]. The structures of the synthesized compounds have been presented in Table 1.

**Table 1:** Scope and yields of synthesized benzothiazepines.

Entry	Structures	Condition/time (h)	Yield %
**R1**	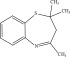	6	76
		8	88
		10	84
**R2**	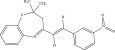	6	73
		8	79
		12	75
**R3**	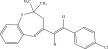	8	78
**R4**	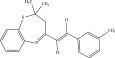	8	76
**R5**	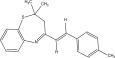	8	75
**R6**	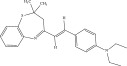	8	72
**R7**	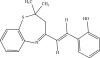	8	77
**R8**	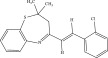	8	81
**R9**	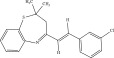	8	76
**R10**	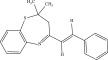	8	80

#### 2.1.1. 2,2,4‐Trimethyl‐2,3‐dihydrobenzo[b][1,4]thiazepine (R1)

A 2‐aminothiophenol (0.03 mol, 3.27 g) was added to 4‐methylpent‐3‐en‐2‐one (0.03 mol, 2.94 g) in methanol, heated under reflux for 8 h and the reaction mixture was allowed to stand overnight in the fume hood. The crude product was recrystallized as a brown solid from ethanol: Yield = 88*%*, Mp = 63^°^C–65^°^C. ^1^H NMR (DMSO‐d6‐400 MHz) *δ* 7.44 (d, *J* = 7.6 *H* *z*, 1H), 7.39 (t, *J* = 7.6 *H* *z*, 1H), 7.07 (m, 2H), 2.23 (s, 2H), 2.22 (s, 3H), 1.43 (s, 6H). ^13^C NMR (DMSO‐*d6*‐100 MHz) *δ* 173.9 (C=N), 152.3 (C), 135.1 (C), 130.0 (CH), 124.9 (CH), 124.4 (C), 63.3 (CH), 47.5 (CH_2_), 31.6 (CH_3_), 29.7 (CH_3_). IR (*ν*max, cm^−1^): 3055 (C–H), 2963 (C–H), 1637 (C=N), 1582 (C=C), 1456 (C–N). Anal. calcd. for C_12_H_15_NS: C, 70.20; H, 7.36; N, 6.82; S, 15.62. Found: C, 70.08; H, 7.23, N, 6.74, S, 15.53. GCMS (m/z, M+): found for C_12_H_15_NS = 205.24, expected mass = 205.32.

#### 2.1.2. (E)‐2,2‐dimethyl‐4‐(3‐nitrostyryl)‐2,3‐dihydrobenzo[b][1,4]thiazepine (R2)

A 3‐nitrobenzaldehye (0.01 mol, 1.51 g) was added to 2,2,4‐trimethyl‐2,3‐dihydrobenzothiazepine (0.01 mol, 2.07 g) in methanol and heated under reflux for 8 h. The reaction mixture was allowed to stand overnight in the fume hood. The crude product was recrystallized and obtained as a brown solid from ethanol: acetone (1:1). Yield = 79*%*, Mp = 122^°^C–124^°^C. ^1^H NMR (DMSO‐d6‐400 MHz) *δ* 8.59 (s, 1H), 8.33 (d, *J* = 7.6 *H* *z*, 1H), 8.28 (t, *J* = 8.0 *H* *z*, 1H), 7.79 (d, *J* = 6.8 *H* *z*, 1H), 7.77 (s, 1H), 7.59 (t, *J* = 7.2 *H* *z*, 1H), 7.53 (t, *J* = 7.2, 8.0 Hz, 1H), 7.43 (s, 1H), 7.39 (s, 1H), 7.21 (m, 1H), 2.66 (s, 2H), 1.54 (s, 6H). ^13^C NMR ((DMSO‐d6‐100 MHz) *δ* 170.1 (C=N), 152.5 (C), 148.8 (C), 138.0 (C), 136.8 (CH), 135.1 (CH), 133.8 (C), 133.5 (C), 130.8 (CH), 130.4 (C), 125.5 (CH), 124.9 (CH), 124.3 (CH), 124.0 (CH), 123.3 (CH), 63.0 (C), 42.0 (CH_2_), 31.1 (C_2_H_6_). IR (*ν*_max_, cm^−1^): 3067 (C–H), 2975 (C–H), 1625 (C=N), 1556 (C=C), 1491 (C–N), 1364 (C–N). Anal. calcd. for C_19_H_20_N_2_O_2_S: C, 67.43; H, 5.36; N, 8.28; S, 9.47, found: C, 67.31; H, 5.24; N, 8.19; S, 9.39. LRMS (*m/z*, M^+^): found for C_19_H_20_N_2_O_2_S = 338.06, expected mass = 338.11.

#### 2.1.3. (E)‐4‐(4‐chlorostyryl)‐2,2‐dimethyl‐2,3‐dihydrobenzo[b][1,4]thiazepine (R3)

4‐Chlorobenzaldehyde (0.01 mol, 1.40 g) was added to 2,2,4‐trimethyl‐2,3‐dihydrobenzothiazepine (0.01 mol, 2.07 g) in methanol and heated under reflux for 8 h. The reaction mixture was allowed to stand overnight in the fume hood. The crude product was recrystallized and obtained as a brown solid from ethanol: acetone (1:1). Yield = 78*%*, Mp = 118^°^C–119^°^C. ^1^H NMR (DMSO‐d6‐400 MHz) *δ* 7.73 (d, *J* = 7.2 *H* *z*, 2H), 7.54–7.44 (m, 4H), 7.19 (s, 1H), 7.13 (t, *J* = 7.2, 8.8 Hz, 3H), 2.55 (s, 2H), 1.44 (s, 6H). ^13^C NMR ((DMSO‐d6‐100 MHz) *δ* 170.3 (C=N), 152.8 (C), 137.7 (CH), 135.2 (C), 135.0 (CH), 134.3 (C), 132.0 (CH), 130.3 (CH), 129.8 (CH), 129.4 (CH), 125.4 (CH), 124.8 (C), 124.3 (C), 62.8 (C), 41.8 (CH_2_), 31.1 (CH_3_). IR (*ν*_max_, cm^−1^): 2976 (C–H), 2964 (C–H), 1625 (C=N), 1556 (C=C), 1491 (C–N), 1406 (C–N), 1364 (C–N). Anal. calcd. for C_19_H_18_ClNS: C, 69.60; H, 5.53; Cl, 10.81; N, 4.27; S, 9.78 found: C, 69.43; H, 5.41; Cl, 10.69; N, 4.22; S, 9.65 LRMS (*m/z*, M^+^): found for C_19_H_18_ClNS = 327.79, expected mass = 327.87.

#### 2.1.4. (E)‐2,2‐dimethyl‐4‐(3‐methylstyryl)‐2,3‐dihydrobenzo[b][1,4]thiazepine (R4)

A 3‐methylbenzaldehyde (0.01 mol, 1.20 g) was added to 2,2,4‐trimethyl‐2,3‐dihydrobenzothiazepin*e* (0.01 mol, 2.07 g) in methanol and heated under reflux for 8 h. The reaction mixture was allowed to stand overnight in the fume hood. The crude product was recrystallized and obtained as a brown solid from ethanol: acetone (1:1). Yield = 76*%*, Mp = 120^°^C–121^°^C. ^1^H NMR (DMSO‐d6‐400 MHz) *δ* 7.51 (m, 5H), 7.32 (m, 1H), 7.21 (m, 1H), 7.12 (m, 3H), 2.56 (s, 2H), 2.35 (s, 3H), 1.46 (s, 6H). ^13^C NMR ((DMSO‐d6‐100 MHz) *δ* 170.4 (C=N), 152.8 (C), 139.3 (CH), 138.6 (C), 136.1 (C), 135.0 (CH), 131.1 (CH), 130.5 (CH), 130.2 (C), 129.3 (CH), 128.8 (CH), 125.3 (CH), 124.8 (CH), 124.3 (CH), 63.8 (C), 41.7 (CH_2_), 31.1 (CH_3_), 21.4 (CH_3_). IR (*ν*_max_, cm^−1^): 3095 (C–H), 3020 (C–H), 2959 (C–H), 2921 (C–H), 2859 (C–H), 1627 (C=N), 1602 (C=C), 1431 (C–N), 1362 (C–N). Anal. calcd. for C_20_H_21_NS: C, 78.13; H, 6.88; N, 4.56; S, 10.43. Found: C, 78.06; H, 6.71; N, 4.56; S, 10.43. LRMS (*m*/*z*, M^+^): found for C_20_H_21_NS = 307.39, expected mass = 307.45.

#### 2.1.5. (E)‐2,2‐dimethyl‐4‐(4‐methylstyryl)‐2,3‐dihydrobenzo[b][1,4]thiazepine (R5)

A 4‐methylbenzaldehyde (0.01 mol, 1.20 g) was added to 2,2,4‐trimethyl‐2,3‐dihydrobenzothiazepine (0.01 mol, 2.07 g) in methanol and heated under reflux for 8 h. The reaction mixture was allowed to stand overnight in the fume hood. The product was recrystallized and obtained as a brown solid from ethanol: acetone (1:1). Yield = 75%, Mp = 128–130^°^C. ^1^H NMR (DMSO‐d6‐400 MHz) *δ* 7.62 (m, 2H), 7.48 (m, 3H), 7.24 (m, 2H), 7.12 (m, 3H), 2.56 (s, 3H), 2.34 (s, 2H), 1.46 (s, 6H). ^13^C NMR ((DMSO‐d6‐100 MHz) *δ* 170.4 (C=N), 152.8 (C), 139.6 (C), 139.1 (CH), 135.1 (CH), 133.5 (C), 130.3 (CH), 130.2 (CH), 130.0 (CH), 128.1 (CH), 125.1 (CH), 124.8 (CH), 124.3 (C), 63.1 (C), 43.0 (CH_2_), 31.1 (CH_3_), 21.5 (CH_3_). IR (*ν*_max_, cm^−1^): 2977 (C–H), 2922 (C–H), 2860 (C–H), 1625 (C=N), 1554 (C=C), 1411 (C–N), 1364 (C–N). Anal. calcd. for C_20_H_21_NS: C, 78.13; H, 6.88; N, 4.56; S, 10.43. Found: C, 78.08; H, 6.76; N, 4.48, S, 10.36. LRMS (*m*/ *z*, M^+^): 307.45. Found for C_20_H_21_NS = 307.40, expected mass = 307.45.

#### 2.1.6. (E)‐4‐(2‐(2,2‐dimethyl‐2,3‐dihydrobenzo[b][1,4]thiazepin‐4‐yl)vinyl)‐N,N‐diethylaniline (**R6**)

*N,N*‐diethylaminobenzaldehyde (0.01 mol, 1.78 g) was added to 2,2,4‐trimethyl‐2,3‐dihydrobenzothiazepine (0.01 mol, 2.07 g) in methanol and heated under reflux for 8 h. The reaction mixture was allowed to stand overnight in the fume hood. The crude product was recrystallized and obtained as a brown solid from ethanol: acetone (1:1). Yield = 72*%*, Mp = 178^°^C–180^°^C. ^1^H NMR (DMSO‐d6‐400 MHz) *δ* 9.77 (s, 1H), 7.74 (d, *J* = 8 *H* *z*, 1H), 7.61 (d, *J* = 8 *H* *z*, 1H), 7.48 (d, *J* = 8 Hz, 2H), 7.42 (t, *J* = 6–8 Hz, 2H), 7.36 (t, *J* = 7.6 Hz, 2H), 7.14 (d, *J* = 7.6 Hz, 2H), 7.08 (t, *J* = 7.2 *H* *z*, 2H), 7.04 (t, J = 7.2 Hz, 2H), 6.97 (s, 2H), 6.93 (d. *J* = 11.6 Hz, 1H), 6.87 (s, 1H), 6.85 (t, *J* = 6–8 Hz, 2H), 1.44 (CH_2_), 1.40 (CH_3_), 1.33 (CH_3_) ^13^C NMR ((DMSO‐d6‐100 MHz) *δ* 191.4 (C), 175.3 (C=N), 170.4 (C), 159.6 (C), 156.0 (C), 153.0 (C), 152.5(C), 139.4 (CH), 135.0 (CH), 134.9 (CH), 134.8 (CH), 132.6 (C), 130.2 (CH), 130.0 (CH), 129.9 (CH), 129.3 (CH), 128.0 (CH), 127.3(CH), 124.9 (CH), 124.8 (CH), 124.7 (CH), 124.2 (CH), 124.1 (CH), 116.4 (C), 116.3 (C), 115.3 (C), 62.8 (C), 62.6 (C), 47.6 (CH_2_), 46.5 (CH_2_), 41.5 (CH_2_), 31.1 (C_2_H_6_). IR (*ν*_max_, cm^−1^): 3054 (C–H), 2960 (C–H), 1603 (C=N), 1578 (C=C), 1548 (C=C), 1362 (C–N). Anal. calcd. for C_23_H_28_N_2_S: C, 75.78; H, 7.74; N, 7.68; S, 8.80. Found: C, 75.67; H, 7.59; N, 7.60; S, 8.71. LRMS (*m*/*z*, M^+^): found for C_23_H_28_N_2_S = 364.48, this the theoretically expected mass of the compound = 364.55.

#### 2.1.7. (E)‐2‐(2‐(2,2‐dimethyl‐2,3‐dihydrobenzo[b][1,4]thiazepin‐4‐yl)vinyl)phenol (R7)

Salicylaldehyde (0.01 mol, 1.22 g) was added to 2,2,4‐trimethyl‐2,3‐dihydrobenzothiazepine (0.01 mol, 2.07 g) in methanol and heated under reflux for 8 h. The reaction mixture was allowed to stand overnight in the fume hood. The crude product was recrystallized and obtained as a brown solid from ethanol: acetone (1:1). Yield = 77*%*, Mp = 160^°^C–161^°^C. ^1^H NMR (DMSO‐d6‐400 MHz) *δ* 10.08 (s, 1H, OH), 7.65 (t, *J* = 7.2, 9.6 Hz, 2H), 7.50 (d, *J* = 7.6 Hz, 1H), 7.44 (t, *J* = 7.2, 7.6 Hz, 1H), 7.18 (m, 2H), 7.10 (t, *J* = 7.6 Hz, 2H), 6.92 (d, *J* = 8.4 Hz, 1H), 6.88 (t, *J* = 7.2, 76, Hz, 1H), 2.52 (s, 2H), 1.45 (s, 6H). ^13^C NMR (DMSO‐d6‐100 MHz) *δ* 170.6 (C=N), 156.6 (C), 153.0 (C), 135.0 (CH), 134.4 (CH), 131.0 (CH), 130.8 (CH), 130.2 (CH), 128.5 (CH), 125.0 (CH), 124.8 (CH), 124.2 (CH), 122.9 (C), 120.0 (CH), 116.6 (CH), 62.9 (C), 42.2 (CH_2_), 31.0 (CH_3_). IR (*ν*_max_, cm^−1^): 2924 (C–H), 2860 (C–H), 1618 (C=N), 1598 (C=C), 1484 (C–N), 1331 (C–N). Anal. calcd. For C_19_H_19_NOS: C, 73.75; H, 6.19; N, 4.53; S, 10.36. Found: C, 73.68; H, 6.08; N, 4.42; S, 10.27. LRMS (*m*/*z*, M^+^): found for C_19_H_19_NOS = 309.36, expected mass = 309.43.

#### 2.1.8. (E)‐4‐(2‐chlorostyryl)‐2,2‐dimethyl‐2,3‐dihydrobenzo[b][1,4]thiazepine (R8)

A 2‐chlorobenzaldehyde (0.01 mol, 1.40 g) was added to 2,2,4‐trimethyl‐2,3‐dihydrobenzothiazepine (0.01 mol, 2.07 g) in methanol and heated under reflux for 8 h. The reaction mixture was allowed to stand overnight in the fume hood. The crude product was recrystallized and obtained as a brown solid from ethanol: acetone (1:1). Yield = 81*%*, Mp = 163^°^C–165^°^C. ^1^H NMR (DMSO‐d6‐400 MHz) *δ* 9.03 (s, 1H), 8.33 (d, *J* = 7.6 Hz, 1H), 8.07 (m, 1H), 7.82 (s, 1H), 7.70 (m), 7.63 (m) 7.51 (m), 7.42 (d, *J* = 8.4), 7.25 (d, *J* = 8.4 Hz), 7.13, 7.10, 7.07, 6.85, 6.81, 2.59, 2.18, 1.57 (CH_3_). ^13^C NMR (DMSO‐d6‐100 MHz) *δ* 174.7 (C=N), 170.3 (C), 152.6 (C), 148. 4 (C), 135.6 (CH), 133.9 (CH), 130.2 (CH), 128.6 (CH), 124.9 (CH), 109.9 (CH), 62.7 (C), 49.7 (C), 46.7 (CH_2_), 42.7 (CH_2_), 31.3 (CH_3_). IR (*ν*_max_, cm^−1^): 3023 (C–H), 2954 (C–H), 1588 (C=C), 1441 (C–N), 1301(C–N). Anal.calcd. for C_19_H_18_ClNS: C, 69.60; H, 5.53; Cl, 10.81; N, 4.27; S, 9.78. Found: C, 69.51; H, 5.47; Cl, 10.72; N, 4.21; S, 9.68. LRMS (*m*/*z*, M^+^): found for C_19_H_18_ClNS = 327.76, expected mass = 327.87.

#### 2.1.9. (E)‐4‐(3‐chlorostyryl)‐2,2‐dimethyl‐2,3‐dihydrobenzo[b] [1, 4] thiazepine (R9)

A 3‐Chlorobenzaldehyde (0.01 mol, 1.40 g) was added to 2,2,4‐trimethyl‐2,3‐dihydrobenzothiazepine (0.01 mol, 2.07 g) in methanol and heated under reflux for 8 h. The reaction mixture was allowed to stand overnight in the fume hood. The crude product was recrystallized and obtained as a brown solid from ethanol: acetone (1:1). Yield = 76*%*, Mp = 121^°^C–122^°^C. ^1^H NMR (DMSO‐d6‐400 MHz) *δ* 7.82 (s, 1H), 7.71 (d, *J* = 6 Hz, 1H), 7.56–7.45 (m, 5H), 7.26 (s, 1H), 7.14 (m, 2H), 2.51 (s, 2H), 1.46. (s, 6H). ^13^C NMR (DMSO‐d6‐100 MHz) *δ* 170.0 (C=N), 152.7 (C), 138.5 (C), 137.5 (CH), 135.0, 134.2 (C), 132.7 (C), 131.2 (CH), 130.2 (CH), 129.4 (CH), 127.9 (CH), 126.6 (CH), 125.4 (CH), 124.9 (CH), 124.3 (C), 63.0 (C), 41.9 (CH_2_), 31.1 (C_2_H_6_). IR (*ν*_masx_, cm^−1^): 3021 (C–H), 2951 (C–H), 1589 (C=C), 1432 (C–N), 1362 (C–N). Anal.calcd. for C_19_H_18_ClNS: C, 69.60; H, 5.53; Cl, 10.81; N, 4.27; S, 9.78. Found: C, 69.52; H, 5.45; Cl, 10.73; N, 4.22; S, 9.69. LRMS (*m*/*z*, M^+^): found for C_19_H_18_ClNS = 327.76, expected mass = 327.87.

#### 2.1.10. (E)‐2,2‐dimethyl‐4‐styryl‐2,3‐dihydrobenzo[b][1,4]thiazepine (R10)

Benzaldehyde (0.01 mol, 1.06 g) was added to 2,2,4‐trimethyl‐2,3‐dihydrobenzothiazepine (0.01 mol, 2.07 g) in methanol and heated under reflux for 8 h. The reaction mixture was allowed to stand overnight in the fume hood. The crude product was recrystallized and obtained as a brown solid from ethanol: acetone (1:1). Yield = 80*%*, Mp = 176^°^C–178^°^C. ^1^H NMR (DMSO‐d6‐400 MHz) *δ* 7.56 (d, *J* = 8 Hz, 2H), 7.48 (d, *J* = 7.2 Hz, 1H), 7.42 (m, 2H), 7.08 (d, *J* = 6.4, 2H), 6.85 (s, 1H), 6.78 (d, *J* = 8 Hz, 2H), 2.53 (s, 2H), 1.45 (s, 6H).^13^C NMR (DMSO‐d6‐100 MHz) *δ* 170.5 (C=N), 153.2 (C), 151.7 (C), 139.9 (CH), 135.0 (CH), 130.3 (CH), 129.6 (CH), 126.1 (CH), 124,7 (CH), 124.2 (C), 123.7 (C), 112.6 (CH), 62.7 (C), 41.7 (CH_2_), 31.0 (CH_3_). IR (*ν*_max_, cm^−1^): 3038 (C–H), 2949 (C–H), 2893 (C–H), 1551 (C=C), 1524 (C=C), 1451 (C–N), 1338 (C–N). Anal. calcd. for C_19_H_19_NS: CC, 77.77; H, 6.53; N, 4.77; S, 10.93. Found: C, 77.68; H, 6.47; N, 4.69; S, 10.81. LRMS (*m*/*z*, M^+^): found for C_19_H_19_NS = 293.35, expected mass = 293.43.

### 2.2. Preparation of Muller Hinton broth

The bacteria were incubated in Muller Hinton broth. Fifty *μ*L of the broth was carefully dispensed into each well of the 96‐well microtitre plate. [30]

### 2.3. Test Organisms

The test organism used in this work was *E. coli* ATCC25922, *S. aureus* (clinical strain), *S. typhi* (ATCC 14028), *K. pneumoniae* NCTC 13440, *C. albicans* (clinical strain), and *E. faecalis* (clinical strain). They were accessed through the School of Basic and Biomedical Sciences Laboratory of the University of Health and Allied Sciences, Ho, Ghana. The test organisms were stored at −20°C until needed; 100 *μ*L of the stock solution was transferred into the nutrient agar and incubated at 37°C for 24 h (subcultured) before use.

### 2.4. Minimum Inhibitory Concentrations (MIC)

The broth micro dilution technique was carried out following the methodology described in document M27‐A3 published by the Clinical and Laboratory Standards Institute (CLSI) (CLSI, 2011 [31]) with minor modifications. Briefly, 50 *μ*L of Mueller Hinton broth was dispensed into all the wells of each of the plates to be used. Fifty microliters of each of the compounds was then used to prepare well concentrations ranging from 30–0.0586 mg/mL (broth with no organism only), respectively, for each microorganism on the columns A–H. Next, 50 *μ*L of each of the 0.5 McFarland standardized test organisms on each column were added after which the plates were subjected to incubation at 37°C for 24 h for the bacterial strains. A bacterial suspension with a final concentration of 5 × 10 cfu/mL was used. The MIC values were evaluated visually by adding 40 *μ*L of 4 mg/mL tetrazolium chloride (TTC) and incubating for 30 min and observed for color change. Moreover, the MIC values of the compounds were cross‐verified by spectrophotometry at 630 nm, employing a microtiter plate reader to ensure precision and consistency.

### 2.5. Determination of Minimum Bactericidal Concentration (MBC)

After determining the MIC, the MBC was determined by reculturing (subculturing) aliquots of 50 *μ*L from the tubes (broth dilution) which show no visible bacterial growth and seeding on MH agar plate and incubated for 24–48 h at 37°C. When 99.9% of the bacterial population is killed at the lowest concentration of an antimicrobial agent. This was done by observing pre and postincubated agar plates for the presence or absence of bacteria [32].

### 2.6. ADMET Analysis

Adsorption, metabolism, excretion, and toxicity studies are all valuable tools in the lengthy process of developing and designing new drugs [33]. The development of computational techniques has greatly aided in the search for novel medications [34]. Some of the challenges in the creation of novel medications are safety and efficacy [35]. Compounds′ properties related to ADMET are crucial to understanding the drug discovery process. A bioactive agent that satisfies the requirements for drug‐likeness is more likely to be developed into a drug if it possesses good ADMET characteristics [36, 37]. When separating apart the characteristics of various compounds, their potential as pharmaceuticals, and their possible modes of administration, Lipinski′s rule of five (RO5) is a useful criterion. The assessment consisted of five criteria: molecular weight (MW) < 500, number of hydrogen bond donors (HBDs) ≤ 5, number of hydrogen bond acceptors (HBAs) ≤ 10, and octanol/water partition coefficient (A log *p*) ≤5 [38]. A compound would not be orally active if it did not meet the requirements or did not comply with two or more of them [39–45]. Table 2 gives the ADMET results for compounds R1 − R10: physicochemical properties (Table 2), medicinal chemistry (Table 3), absorption (Table 4), distribution (Table 5), metabolism (Table 6), excretion (Table 7), toxicity (Table 8), environmental toxicity (Table 9), tox21 pathway (Table 10).

**Table 2:** Molecular properties of compound R1–R10: (A) physicochemical properties.

(A) physicochemical properties											
Property	R1	R2	R3	R4	R5	R6	R7	R8	R9	R10	Ampicillin
Molecular weight	205.090	338.110	327.080	307.140	307.140	364.200	309.120	327.080	327.08	293.120	349.110
Volume	217.955	345.866	335.136	337.221	337.221	400.106	328.715	335.136	335.136	319.925	330.46
Density	0.941	0.978	0.976	0.911	0.911	0.910	0.940	0.976	0.976	0.916	1.056
NHA	1	4	1	1	1	2	2	1	1	1	7
NHD	0	0	0	0	0	0	1	0	0	0	4
nRot	0	3	2	2	2	5	2	2	2	2	5
NRing	2	3	3	3	3	3	3	3	3	3	3
MaxRing	11	11	11	11	11	11	11	11	11	11	7
nHet	2	5	3	2	2	3	3	3	3	2	8
fChar	0	0	0	0	0	0	0	0	0	0	0
nRing	12	20	19	19	19	19	19	19	19	19	17
Flexibility	0.000	0.150	0.105	0.105	0.105	0.263	0.105	0.105	0.105	0.105	0.294
Stereo Centers	0	0	0	0	0	0	0	0	0	0	4
TPSA	12.360	55.500	12.360	12.360	12.360	15.600	32.590	12.360	12.360	12.360	112.730
log S	−2.443	−5.496	−5.839	−5.638	−5.659	−6.641	−4.908	−5.754	−5.796	−5.166	−1.564
log P	2.534	4.184	5.113	4.810	4.863	5.577	4.346	4.946	5.052	4.383	0.899
log D	2.928	4.076	4.041	4.069	4.073	4.432	3.855	4.062	4.038	3.991	0.589

**Table 3:** (B) medicinal chemistry.

B. Medicinal chemistry											
Property	R1	R2	R3	R4	R5	R6	R7	R8	R9	R10	Ampicillin
QED	0.622	0.536	0.621	0.657	0.657	0.594	0.799	0.621	0.621	0.687	0.675
Synthetic accessibility score	2.959	2.885	2.764	2.816	2.748	2.883	2.907	2.831	2.829	2.726	3.527
Fsp3	0.417	0.211	0.211	0.250	0.250	0.348	0.211	0.211	0.211	0.211	0.438
MCE‐18	26.824	39.130	36.957	36.720	36.720	38.323	36.957	36.957	36.957	34.783	70.304
NPscore	−0.331	−0.667	−0.366	−0.324	−0241	−0.555	0.092	−0.583	−0.484	−0.082	0.742
Lipinski′s rule	Yes	Yes	Yes	Yes	Yes	Yes	Yes	Yes	Yes	Yes	Yes
Pfitzer	Yes	No	No	No	No	No	No	No	No	No	Yes
GSK rule	Yes	No	No	No	No	No	No	No	No	No	Yes
Golden triangle	Yes	Yes	Yes	Yes	Yes	Yes	Yes	Yes	Yes	Yes	Yes

**Table 4:** (C). Absorption.

C. Absorption											
Property	R1	R2	R3	R4	R5	R6	R7	R8	R9	R10	Ampicillin
Caco‐2 permeability	−4.643	−4.674	−4.745	−4.805	−4.774	−4.790	−4.777	−4.786	−0.4779	−0.4721	−5.910
MDCK permeability	2.7 e‐05	6.4e‐05	1 e‐0.5	1.6e‐05	1.6 e‐05	1.6e‐05	1.9 e‐05	1.3e‐05	9.9 e‐06	1.6 e‐05	3.1e‐0.5
Pgp inhibitor	Excellent	Excellent	Excellent	No	No	No	Excellent	Excellent	Excellent	Excellent	Excellent
Pgp substrate	Excellent	Excellent	Excellent	Excellent	Excellent	Excellent	Excellent	Excellent	Excellent	Excellent	Excellent
Human intestinal absorption	Excellent	Excellent	Excellent	Excellent	Excellent	Excellent	Excellent	Excellent	Excellent	Excellent	Excellent
Human oral bioavailability 20%	Excellent	Excellent	Excellent	Excellent	Excellent	Excellent	Excellent	Excellent	Excellent	Excellent	Excellent
Human oral bioavailability 30%	Excellent	Excellent	Excellent	Excellent	Excellent	Excellent	Excellent	Excellent	Excellent	Excellent	Excellent

**Table 5:** (D). Distribution.

D. Distribution											
Property	R1	R2	R3	R4	R5	R6	R7	R8	R9	R10	Ampicillin
Plasma protein binding	85.19%	98.66%	98.41%	97.87%	97.76%	97.91%	97.79%	98.39%	98.79%	97.32%	48.54%
Volume Distribution.	4.67	0.82	1.32	0.91	1.07	2.17	0.940	1.702	1.525	1.144	0.222
Blood–brain barrier Penetration	Yes	Yes	Yes	Yes	Yes	No	Yes	Yes	Yes	Yes	No
Fraction unbound	15.63%	1.15%	1.75%	2.09%	2.47%	1.94%	1.90%	1.94%	1.56%	2.73%	67.51%

**Table 6:** (E). Metabolism.

E. Metabolism											
Property	R1	R2	R3	R4	R5	R6	R7	R8	R9	R10	Ampicillin
CYP1A2 inhibitor	Yes	Yes	Yes	Yes	Yes	Yes	Yes	Yes	Yes	Yes	No
CYP1A2 substrate	Yes	No	Yes	Yes	Yes	Yes	Yes	Yes	Yes	Yes	No
CYP2C19 inhibitor	No	Yes	Yes	Yes	Yes	Yes	Yes	Yes	Yes	Yes	No
CYP2C19 Substrate	Yes	No	No	No	Yes	No	No	No	No	No	No
CYP2C9 inhibitor	No	Yes	Yes	No	Yes	Yes	Yes	Yes	Yes	Yes	No
CYP2C9 substrate	Yes	Yes	Yes	Yes	Yes	Yes	Yes	Yes	Yes	Yes	No
CYP2D6 inhibitor	No	Yes	Yes	Yes	Yes	Yes	Yes	Yes	Yes	Yes	No
CYP2D6 substrate	Yes	Yes	Yes	Yes	Yes	Yes	Yes	Yes	Yes	Yes	No
CYP3A4 Inhibitor	No	Yes	No	Yes	Yes	Yes	Yes	Yes	No	No	No
CYP3A4 substrate	No	Yes	Yes	Yes	Yes	Yes	Yes	Yes	Yes	Yes	No

**Table 7:** (F). Excretion.

F. Excretion											
Property	R1	R2	R3	R4	R5	R6	R7	R8	R9	R10	Ampicillin
CL	5.526	1.926	2.335	3.202	2.992	4.749	3.114	2.561	2.617	2.893	3.376
T_1/2_	0.390	0.095	0.060	0.118	0.090	0.095	0.226	0.057	0.074	0.122	0.975

**Table 8:** (G). Toxicity.

G. Toxicity											
Property	R1	R2	R3	R4	R5	R6	R7	R8	R9	R10	Ampicillin
hERG Blockers	Excellent	Excellent	Excellent	Excellent	Excellent	Excellent	Excellent	Excellent	Excellent	Excellent	Excellent
Human hepatotoxicity	Poor	Excellent	Excellent	Excellent	Excellent	Excellent	Excellent	Excellent	Excellent	Excellent	Excellent
Drug‐induced liver injury	Poor	Poor	Poor	Poor	Poor	Poor	Poor	Poor	Poor	Poor	Poor
AMES toxicity	Excellent	Poor	Poor	Poor	Poor	Poor	Poor	Poor	Poor	Poor	Excellent
Rat oral acute toxicity	Excellent	Excellent	Excellent	Excellent	Excellent	Excellent	Excellent	Excellent	Excellent	Excellent	Excellent
FDAMDD	Excellent	Poor	Excellent	Excellent	Excellent	Excellent	Excellent	Excellent	Excellent	Excellent	Poor
Skin Sensitization	Poor	Poor	Poor	Poor	Poor	Poor	Poor	Poor	Poor	Poor	Excellent
Carcinogenicity	Poor	Poor	Poor	Poor	Poor	Poor	Poor	Poor	Poor	Poor	Excellent
Eye corrosion	Excellent	Excellent	Excellent	Excellent	Excellent	Excellent	Excellent	Excellent	Excellent	Excellent	Excellent
Eye irritation	Poor	Poor	Poor	Poor	Poor	Poor	Poor	Poor	Poor	Poor	Excellent
Respiratory toxicity	Excellent	Poor	Excellent	Excellent	Excellent	Poor	Excellent	Excellent	Excellent	Excellent	Excellent

**Table 9:** (H). Environmental toxicity.

H. Environmental toxicity											
	R1	R2	R3	R4	R5	R6	R7	R8	R9	R10	Ampicillin
Biocentration Factors	2.544	2.858	3.156	2.936	2.923	2.558	2.360	3.041	3.142	2.865	0.356
IGC50	3.737	5.032	5.134	5.040	5.047	5.192	5.077	5.141	5.148	4.959	2.602
LC50FM	4.230	6.061	6.194	5.831	5.912	6.331	5.661	6.030	6.136	5.811	3.589
LC50DM	4.477	5.897	6.170	5.858	5.833	6.624	5.776	6.018	6.042	5.651	3.860

**Table 10:** (I). Tox21 pathway.

I. Tox21 pathway											
Property	R1	R2	R3	R4	R5	R6	R7	R8	R9	R10	Ampicillin
Androgen receptor	Inactive	Inactive	Inactive	Inactive	Inactive	Inactive	Inactive	Inactive	Inactive	Inactive	Inactive
Androgen receptor ligand binding domain (AR‐LBD)	Inactive	Active	Inactive	Inactive	Inactive	Inactive	Active	Active	Inactive	Inactive	Inactive
Aryl hydrocarbon receptor (AhR)	Inactive	Inactive	Inactive	Inactive	Inactive	Active	Active	Inactive	Inactive	Inactive	Inactive
Aromatose	Inactive	Active	Active	Inactive	Active	Active	Active	Active	Active	Active	Inactive
Estrogen receptor alpha (ER)	Inactive	Active	Active	Active	Active	Active	Active	Active	Active	Active	Inactive
Estrogen receptor ligand binding domain ER‐LBD	Inactive	Active	Inactive	Inactive	Inactive	Inactive	Active	Active	Inactive	Inactive	Inactive
Peroxisome proliferator activated receptor gamma (PPAR‐gamma)	Inactive	Active	Active	Active	Active	Active	Active	Active	Active	Active	Inactive
SR‐ARE	Inactive	Active	Active	Active	Active	Active	Active	Active	Active	Active	Inactive
SR‐ATAD5	Inactive	Active	Active	Active	Active	Active	Active	Active	Active	Active	Inactive
Heat shock factor response element (HSE)	Inactive	Active	Active	Active	Active	Active	Active	Active	Active	Active	Inactive
Mitochondrial membrane protein	Inactive	Active	Active	Active	Active	Active	Active	Active	Active	Active	Inactive
Phosphoprotein (tumor suppressor) p53	Inactive	Active	Active	Inactive	Inactive	Active	Active	Active	Active	Inactive	Inactive

### 2.7. Molecular Docking

The docking study was performed on a computer system equipped with Ubuntu 22.04.3 LTS, an 11th Gen Intel Corei7‐11800H @ 2.30GHz × 16 processor, 32.00 GB installed RAM, GeForce RTX 3060 NVIDIA and a 64‐bit operating system. The molecular docking analysis used five target proteins from Gram (+ve) *S. aureus* and Gram (‐ve) *E. coli*, which include, transpeptidase (Protein Data Bank [PDB] ID: 5TW8 and 6NTW), DNA gyrase B subunit (PDB ID: 4URN and 1KZN), dihydrofolate reductase (PDB ID: 6XG5 and 6P9Z), dihydropteroate synthase (DHPS) (PDB ID: 1 AD4 and 5V7A), and muramyl ligase (PDB ID: 4C13 and 1E8C) to investigate the potential mechanism of action of the small‐molecule compounds as antibacterial agents. The existing research indicates that these proteins are crucial for the survival of the corresponding bacteria [46]. Therefore, inhibition of any of the proteins can destroy these microorganisms by getting in the way of their nucleic acid cleavage, assembly, and replication, or by disrupting the components and functions of their cell walls. We obtained the crystal structure of the proteins from the PDB and processed it using the Protein Preparation Wizard of Schrödinger Maestro 13.5 version. The 2D and 3D coordinates of the ligands were generated using the ChemDraw2D/3D software. The ligands were prepared and optimized for docking using the Ligprep module of Schrödinger Maestro 13.5 version [47]. The receptor grid was developed using the glide application. An enclosed box was generated within the prepared protein structure by selecting any atom of the cocrystallized ligand from the protein′s binding site, followed by docking. The best poses of the compounds were postprocessed with prime MM‐GBSA [48].

## 3. Results

### 3.1. Synthesis and Characterization

The compounds were obtained by heating the reagents in methanol for a minimum of 6 h under reflux. Scheme 1 gives a synthetic scheme for the synthesis of the 2,3‐dihydrobenzo[b] [1, 4] thiazepine derivatives. 2,2,4‐Trimethyl‐2,3‐dihydrobenzo[b] [1, 4] thiazepine (1), which was the starting material for all the other derivatives was synthesized by the reaction of 2‐aminothiophenol with 4‐methylpent‐3‐en‐2‐one. Thin‐layer chromatography was used to follow the reaction to see the disappearance of the spot for the starting material. Compound R1 was reacted with different aldehydes in methanol to give the final products. The synthesized compounds are listed in Table 1. Figures S1–S40 contain the IR and NMR data used for characterizing the compounds.

**Scheme 1:**
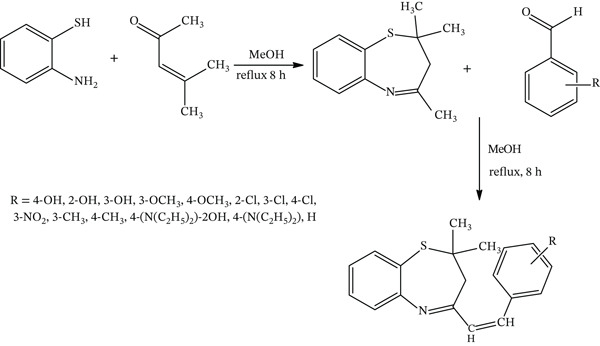
Reaction scheme for 2,3‐dihydrobenzo[b][1,4]thiazepine derivatives.

**Scheme 2:**
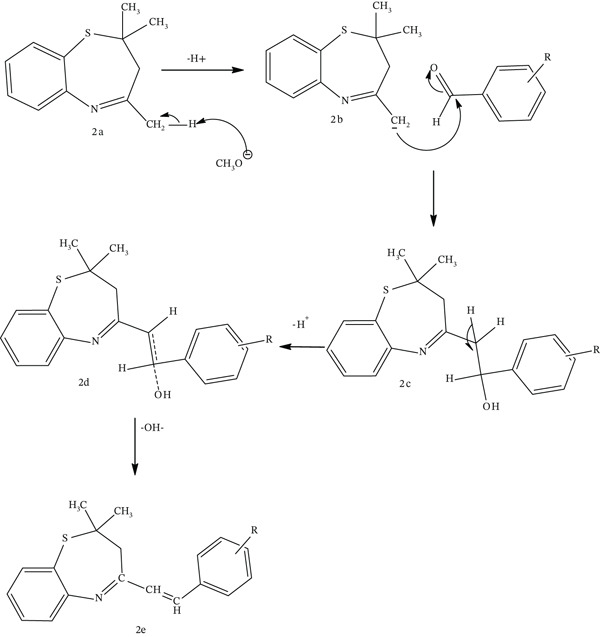
Reaction mechanism for the synthesis of 2,3‐dihydrobenzo[b][1,4]thiazepine derivatives.

Scheme 2 gives the proposed reaction mechanism for the synthesis of 2,3‐dihydrobenzo[b][1,4]thiazepine derivatives. It is proposed that the reaction proceeds by a proton abstraction from the methyl group connected to the imine on the benzothiazepine ring by the methoxide ions in solution (2a); a proton loss yields a carbanion which attacks the aldehydic carbonyl (2b), forming a hydroxyl group (2c). A methylene proton loss leads to the intermediate (2d), which subsequently loses water to form the final product. This mechanism is similar to that proposed for the benzoxazepine and benzodiazepine derivatives synthesized by a similar method [49, 50].

The infrared spectrum of compound R1 showed the presence of characteristic absorption peaks at 3055 and 2963 cm^−1^ for the C–H stretch, respectively. Peaks were observed at 1637, 1582 and 1456 cm^−1^ for the C=N, C=C, and C–N absorptions, respectively. Compound R1 has been reported in our earlier work [51]. For compounds R1–R10 (Table S1 in the Supporting Information), the C–H stretch appears in the range 3095−2859 cm^−1^, whereas aliphatic C=N, C=C, and C‐N stretches lie in the range 1637−1603, 1602−1548 and 1491−1301 cm^−1^, respectively.

The ^1^H NMR spectra of the synthesized compounds indicated the presence of the methyl groups on the benzothiazepine ring between 1.33 and 1.54 ppm, which were confirmed in the ^13^C NMR and DEPT spectra between 21.5 and 31.3 ppm. The incorporation of the methylene group was confirmed in all the compounds in the ^1^H NMR spectra between 2.18 and 2.66 ppm. This was also confirmed in the ^13^C NMR and DEPT spectra between 41.8 and 47.6 ppm. The ^1^H NMR of compound R1 confirmed formation of the benzothiazepine ring with the formation of a methyl group and two methyl groups at 2.23 and 1.43 ppm, respectively. The incorporation of the methylene group was observed at 2.34 ppm. This was confirmed in the ^13^C NMR and DEPT spectra at 47.5 ppm, which also gave signals for the methyl groups at 31.6 and 29.7 ppm.

The ^1^H NMR spectra for compound R2 confirmed the presence of the methylene group at 2.66 ppm and two methyl groups at 1.54 ppm. Peaks confirming the incorporation of the C=N were observed at 173.9 and 170.1 ppm for compounds R1 and R2, respectively, in the ^13^C NMR spectrum. A singlet signal was observed at 8.59 ppm for the aromatic proton next to the 3‐nitro group, which confirms the incorporation of the 3‐nitrobenzaldehyde moiety on the benzothiazepine ring. The ^1^H NMR spectrum for compound R3 confirmed the presence of the methylene group and two methyl groups at 2.55 and 144 ppm, respectively, whereas the C=N signal was observed at 170.3 ppm in the ^13^C NMR spectrum. The presence of methyl groups at 2.35 and 31.1 ppm in the ^1^H NMR and ^13^C NMR spectra confirmed the incorporation of the 3‐methylbenzaldehyde onto the benzothiazepine ring in compound R4. Signals for the methylene protons and the methyl protons of the benzothiazepine ring were observed at 2.56 and 1.44 ppm in the ^1^H NMR spectrum which were confirmed in the ^13^C NMR spectrum at 41.7 and 21.4 ppm, respectively. The ^1^H NMR spectrum of compound R5 confirmed the incorporation of the 4‐methylbenzaldehyde by a signal at 2.56 ppm which was confirmed in the ^13^C NMR at 31.1 ppm. The ^1^H NMR and ^13^C NMR spectra of compound R6 confirmed the presence of two molecules of the compound, one of which is protonated resulting in a doubling of all the signals in the spectrum. The incorporation of diethylamine was confirmed by the methylene groups at 47.78 and 46.47 ppm in the ^13^C NMR spectrum. The ^1^H NMR spectrum of compound R7 confirmed the presence of the hydroxyl proton at 10.08 ppm, which indicated that the salicylaldehyde moiety was successfully incorporated. The ^1^H NMR spectra of compounds R8 and R9 confirmed the incorporation of the 2‐chlorobenzaldehyde and 3‐chlorobenzaldehyde onto the benzothiazepine ring with signals for the methylene protons observed at 2.18 and 2.51 ppm, whereas peaks for the methyl protons were observed at 1.57 and 1.46 ppm, respectively. In the ^13^C NMR spectrum, the methylene groups were observed at 46.7 and 41.9 ppm, respectively, whereas the methyl groups were observed at 31.3 and 31.1 ppm, respectively.

### 3.2. Antimicrobial Activity: Minimum Inhibitory/Bactericidal Concentrations (MICs/MBCs)

The MICs, MBCs, and synergistic potentials of the compounds (benzothiazepines) were measured in order to examine their range of action against various bacterial strains. A dose‐dependent method was used to calculate the MICs. Most benzothiazepines exhibited potent bactericidal action, with MIC values ranging from 1.88 to 15 mg/mL (Tables 11, 12, 13, and 14). Compound R9 demonstrated the least efficacy against *E. feacalis*, whereas compounds R4 and R6 demonstrated the most activity. Compound 1 had the highest activity against *E. coli*, with a MIC of 7.5 mg/mL, whereas compound R6 had the lowest activity, with a MIC of 15 mg/mL.

Compounds R5 and R9 had the lowest activity, with MIC values of 15 mg/mL, whereas compound R6 was the most effective against S. aureus, with a MIC of 1.875 mg/mL. The MBCs for the benzothiazepines demonstrated that the compounds were bactericidal against the test strains utilized in addition to the MIC determination, with the exception of compounds R2 and R10, which displayed no activity. This is consistent with research showing that benzodiazepines′ antimicrobial action against microorganisms can be categorized as either strong (MIC <2 mg/mL) or good (MIC range of 2–10 mg/mL). An MBC/MIC ratio of ≤ 4 indicates a bactericidal agent, whereas an MBC/MIC ratio of > 4 indicates a bacteriostatic agent [51–53]. The minimum bactericidal/fungicidal concentration and MBC/MIC ratios for the evaluated compounds against the selected bacteria are shown in Tables 11, 12, 13, and 14 **[** 54].

**Table 11:** MIC, MBC, and MBC/MIC for compounds R1 and R3.

Organism	R1			R3		
	MIC (mg/mL)	MBC (mg/mL)	MBC/MIC	MIC (mg/mL)	MBC (mg/mL)	MBC/MIC
*S. aureus*	15.00	15.00	1	3.75	15.00	4
*E. feacalis*	7.50	7.50	1	3.75	15.00	4
*E. coli*	7.50	Null	N.D	Null	Null	N.D
*K. pneumonae*	15.00	15.00	1	3.75	15.00	4

**Table 12:** MIC, MBC, and MBC/MIC for compounds R4 and R5.

Organism	R4			R5		
	MIC (mg/mL)	MBC (mg/mL)	MBC/MIC	MIC (mg/mL)	MBC (mg/mL)	MBC/MIC
*S. aureus*	3.75	7.50	2.00	15.00	15.00	1.00
*E. feacalis*	1.875	Null	N.D	3.75	7.50	2.00
*E. coli*	Null	Null	N.D	Null	Null	N.D
*K. pneumonae*	5.63	15.00	2.67	11.25	Null	ND

**Table 13:** MIC, MBC, and MBC/MIC for compounds R6 and R7.

Organism	R6			R7		
	MIC (mg/mL)	MBC (mg/mL)	MBC/MIC	MIC (mg/mL)	MBC (mg/mL)	MBC/MIC
*S. aureus*	1.875	7.50	4.00	7.50	7.50	1.00
*E. feacalis*	1.875	3.75	2.00	7.50	7.50	1.00
*E. coli*	15.00	15.00	1.00	Null	Null	N.D
*K. pneumonae*	7.50	7.50	1.00	7.50	15.00	2.00

**Table 14:** MIC, MBC, and MBC/MIC for compounds R8 and R9.

Organism	R8			R9		
	MIC (mg/mL)	MBC (mg/mL)	MBC/MIC	MIC (mg/mL)	MBC (mg/mL)	MBC/MIC
*S. aureus*	11.25	15.00	1.33	15.00	15.00	1.00
*E. feacalis*	7.50	7.50	1.00	11.25	7.50	0.667
*E. coli*	Null	Null	N.D	Null	Null	N.D
*K. pneumonae*	11.25	Null	ND	15.00	15.00	1.00

Abbreviation: ND, not determined.

### 3.3. Molecular Docking

**Figure 1:**
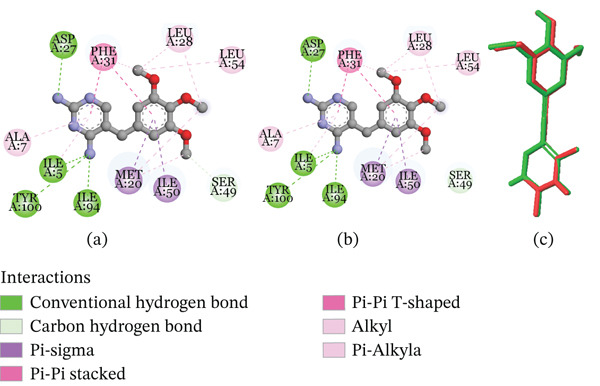
Validation of docking protocol: interaction between cocrystallized ligand (trimethoprim) and the protein structure (6XG5), (a) before redocking procedure and (b) after redocking procedure. (c) Superimposition: cocrystal (red color) and docked best pose (green color).

Molecular docking is a widely used computational method in structure‐based drug design. It is used to predict the binding poses of candidate compounds and differentiate binders from nonbinders in a defined binding pocket [55]. Prior to conducting the docking experiments, we utilized Maestro software to examine the type of connections between the cocrystallized ligands and the protein active site residues. This step was critical because it provides valuable information about how the cocrystallized ligand binds and interacts with key residues. These interactions include hydrogen bond, halogen bond, *π*‐cation, *π*–*π* stacking, salt bridge, and metal coordination with the catalytic and nearby catalytic residues. In order to validate the docking protocol, we performed a redocking experiment using one of the protein structures and its cocrystallized ligand. Subsequently, we compared the interaction information between the redocked ligand and the protein with the original ligand plot information obtained from the protein databank. Figure 1 shows similar binding between the cocrystallized ligand and the protein structure, both before and after the redocking procedure. The root mean square deviation (RMSD) value from the redocking experiment was also convincing, with all the redocked poses having RMSD values between 0.05 Å and 0.2 Å. Figure 1 depicts the ligand superimposition: cocrystal ligand (red color) and redocked pose (green color). Redocked poses with RMSD values below 2.0 are indicative of higher dependability [56]. This is because lower values are more favorable and correspond to more reliable docking results.

**Figure 2:**
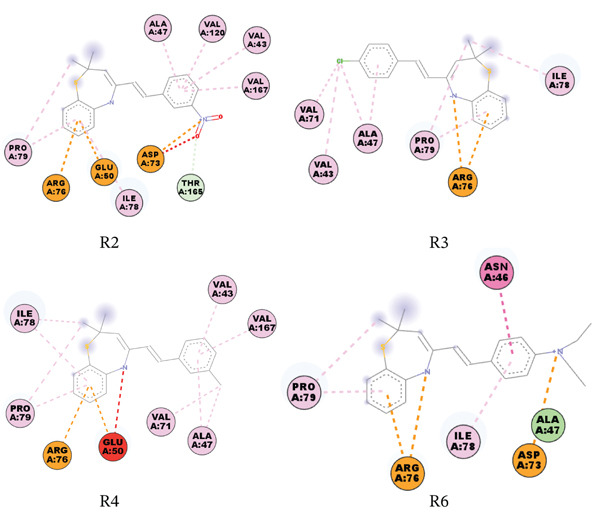
Interaction network between DNA gyrase B subunit of *E. coli* (PDB ID: 1KZN) and the studied compounds.

**Figure 3:**
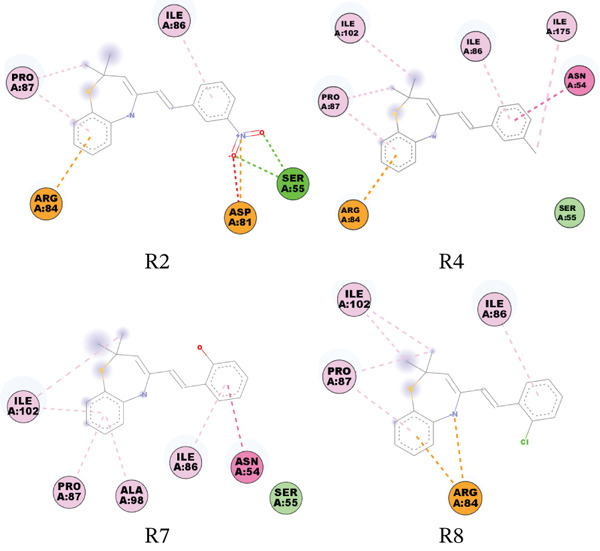
Interaction network between DNA gyrase B subunit of *S. aureus* (PDB ID: 4URO) and the studied compounds.

**Figure 4:**
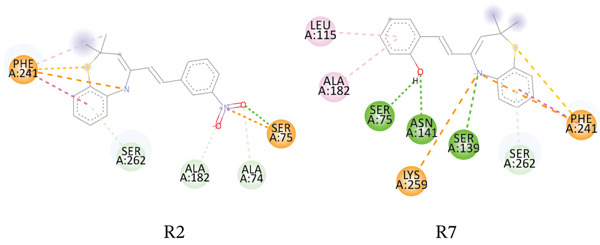
Interaction network between *S. aureus* transpeptidase (PDB ID: 5TW8) and the studied compounds.

**Figure 5:**
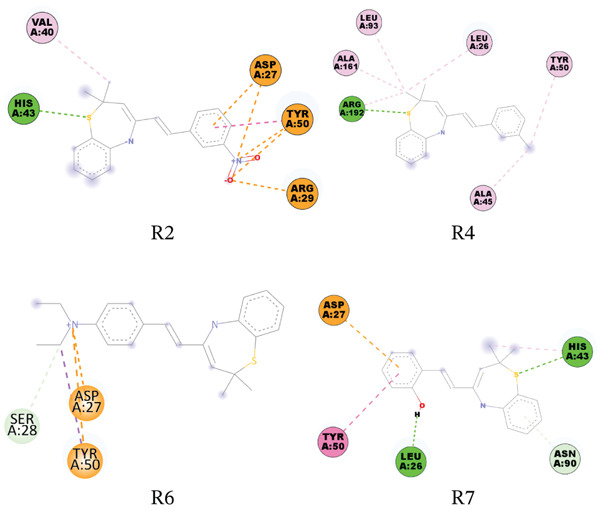
Interaction network between *E. coli* muramyl ligase E (PDB ID: 1E8C) and the studied compounds.

**Figure 6:**
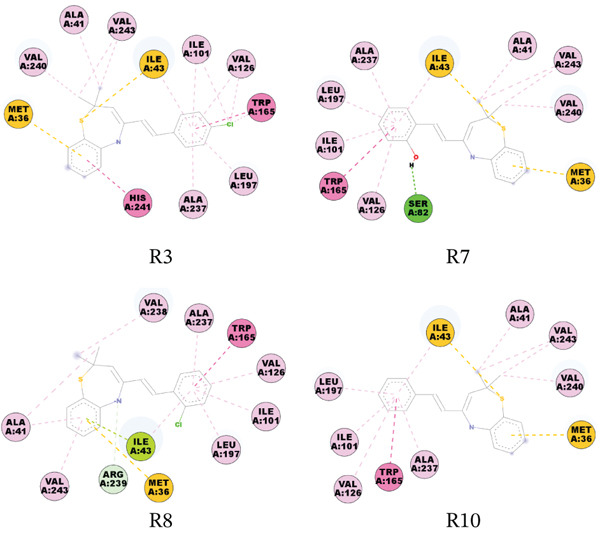
Interaction network between *S. aureus* DHPS (PDB ID: 1 AD4) and the studied compounds.

The molecular docking study revealed that the DNA gyrase B subunit of *E. coli* had binding energies of −1.49 to −3.98 kcal/mol. Compound R2 binds to this target with a binding affinity score of −3.27 kcal/mol and forms one salt bridge with ASP73; compound R3 binds with a binding affinity score of −3.15 kcal/mol and forms one halogen bond with VAL71; and compound R6 binds with a binding affinity score of −2.09 kcal/mol and forms one *π*‐cation and one salt bridge with ARG76 and ASP73, respectively. Figures 2, 3, 4, 5, and 6 give the 2D pictorial view of the best performing compounds against different protein targets.

The remaining compounds seem not to make significant interactions with the active site residues at the 4.0 Å cutoff. The compounds′ binding affinity scores to the DNA gyrase B subunit of *S. aureus* were not significantly different from those of *E. coli*. The scores were lower compared with the control drug (ampicillin). In terms of interaction type, the compounds form *π*‐cation interaction with ASP81 and ARG84.

Ampicillin seems to bind more favorably to this target than the compounds. It had a binding affinity score of −5.94 kcal/mol on the DNA gyrase B subunit of *E. coli* and −5.59 kcal/mol on the DNA gyrase B subunit of *S. aureus*. Ampicillin also forms at least two hydrogen bonds, as well as a salt bridge, *π*‐cation, and *π*–*π* stacking with active site residues. Furthermore, we observed binding affinities ranging from approximately −2.09 to −4.0 kcal/mol and −1.31 to −3.85 kcal/mol against the bacterial transpeptidase of *S. aureus* and *E. coli*, respectively. For both proteins, compound R2 had a binding affinity score of about ‐3.85 kcal/mol; however, it only formed a salt bridge with SER75 of *S. aureus* transpeptidase. Compound R7 had a binding affinity score of about −4.0 kcal/mol on the *S. aureus* transpeptidase, connecting with SER75 and ASN141 in two H‐bonds and a *π*–*π* stacking with PHE241. The compounds mentioned above exhibit a moderate interaction with *S. aureus* transpeptidase in comparison to ampicillin.

All other compounds have a poor to mild binding and interaction with this target′s active site residue. The study also revealed that the binding energies of muramyl ligase E (MurE) from *S. aureus* and *E. coli* bacteria ranged from −1.59 to −2.84 kcal/mol and −1.79 to −3.39 kcal/mol, respectively. MurE is one of the amide ligases that catalyzes the synthesis of nonribosomal peptide bonds, which are necessary for the attachment of the peptide moiety to the peptidoglycan building blocks [53]. As shown in Table 15, all the compounds exhibited poor binding towards the *S. aureus* muramyl ligase. Compounds R2, R6, and R7, however, showed a mild binding affinity to the *E. coli* muramyl ligase E. Ampicillin demonstrated better binding to this target in comparison to all the compounds.

**Table 15:** Docking results (kcal/mol)) estimated for all the compounds.

	DNA gyrase B		Transpeptidase		Muramyl ligase E		DHPS		DHFR	
Compound	4URO	1KZN	5TW8	6NTW	4C13	1E8C	1 AD4	5V7A	6XG5	6P9Z
**R1**	−2.52	−1.49	−3.17	−2.84	−2.1	−2.85	−5.18	−2.8	−3.67	−3.48
**R2**	−2.56	−3.27	−3.85	−3.85	−1.82	−3.25	−3.98	−2.58	−5.87	−4.71
**R3**	−2.15	−3.15	−3.33	−2.95	−1.93	−2.09	−5.03	−3.99	−4.66	−4.7
**R4**	−3.58	−3.98	−3.34	−1.76	−1.93	−2.98	−5.21	−4.04	−4.67	−5.29
**R5**	−1.99	−3.66	−3.05	−3.21	−1.94	−2.81	−5.6	−2.75	−4.55	−5.45
**R6**	−2.7	−2.09	−2.09	−1.31	−1.59	−3.09	−5.5	−2.24	−4.51	−5.05
**R7**	−2.56	−3.64	−4	−2	−2.5	−3.39	−5.94	−3.46	−5	−5.17
**R8**	−2.77	−3.66	−2.81	−3.33	−2.08	−2.99	−5.77	−2.6	−5.41	−5.37
**R9**	−2.6	−3.85	−2.98	−2.86	−1.86	−2.95	−4.71	−2.54	−5.04	−4
**R10**	−2.41	−3.68	−2.86	−2.88	−2.84	−2.78	−5.07	−2.6	−4.64	−4.66
**Ampicillin**	−5.59	−5.94	−4.14	−4.131	−6.14	−6.3	−6.47	−5.92	−8.34	−5.85

The compounds strongly bind to DHPS targets, especially *S. aureus* DHPS with binding affinity scores ranging from −3.98 kcal/mol to −5.94 kcal/mol. Hydrogen bonding with SER82 and pi–pi stacking with TRP165 were the most prevalent modes of binding the compounds to this protein.

DHPS, which catalyzes the condensation of p‐aminobenzoic acid (pABA) with 7,8‐dihydro‐6‐hydroxymethylpterin pyrophosphate (DHPt‐PP) to form 7,8‐dihydropteroate and pyrophosphate, is a known druggable target in de novo synthesis in microbial cells [57]. DHPS has been used as a drug target, with sulfonamides and sulfone derivatives like sulfamethoxazole being used as antimicrobial agents [58]. Sulfonamides, structural analogs of pABA, act as alternative substrates for DHPS, resulting in sulfapterines that cannot be used in the biosynthetic pathway. The dihydrofolate reductase enzyme (DHFR) was the final druggable bacterial target we considered in this study. This enzyme catalyzes the reduction of 5,6‐dihydrofolic acid (DHF) to 5,6,7,8‐tetrahydrofolic acid (THF) by enantiospecific hydride transfer from the NADPH cofactor [59]. THF and its derivatives are essential precursors for the biosynthesis of DNA bases and amino acids. We found that this target in both *E. coli* and *S. aureus* had the strongest binding affinity for all the compounds, with binding affinity scores ranging from −3.67 kcal/mol to −5.87 kcal/mol.

To further confirm our findings, we postprocessed the best docked poses with prime molecular mechanics generalized Born surface area (MM/GBSA), which is a popular and rigorous method to calculate the free energy of the binding of ligands to proteins. End‐point–free energy methods, based on sampling of the final states of a system, are less expensive and more accurate than most docking scoring functions. The results, as shown in Table 16, agreed with the molecular docking analysis.

**Table 16:** Prime MMGBSA dG Bind of the study compounds.

Compound	DNA gyrase B		Transpeptidase		Muramyl ligase E		DHPS		DHFR	
	4URO	1KZN	5TW8	6NTW	4C13	1E8C	1 AD4	5V7A	6XG5	6P9Z
**R1**	−24.88	−20.35	−13.14	−12.1	−15.61	−10.42	−31.19	−16.79	−18.08	−32.44
**R2**	−24.22	−32.7	−21.76	−30.48	−17.46	−43.3	−17.33	−16.71	−50.11	−41.72
**R3**	−17.43	−35.77	−22.06	−28.95	−19.69	−27.27	−40.66	−16.33	−49.62	−46.72
**R4**	−22.57	−34.33	−14.23	−21.4	−18.24	−27.01	−35.15	−13.36	−45.77	−40.91
**R5**	−22.58	−31.33	−14.87	−27.48	−19.11	−24.04	−33.69	−11.74	−45.25	−46.15
**R6**	−22.24	−28.99	−4.92	−35.94	−23.06	−48.4	−34.71	−8.56	−34.44	−31.52
**R7**	−19.45	−29.62	−9.95	−14.28	−19.98	−50.64	−46.88	−24.18	−30.18	−46.2
**R8**	−22.29	−27.9	−10.04	−27.91	−22.31	−27.84	−18.59	−13.11	−48.48	−30.47
**R9**	−22.01	−34.8	−16.23	−29.05	−10.49	−40.25	−18.76	−10	−48.18	−48.29
**R10**	−18.38	−28.87	−13.26	−25.3	−25.43	−37.16	−42.6	−12.54	−41.67	−46.55
**Ampicillin**	−38.71	−31.13	−32.63	−23.48	−20.81	−31.39	−38.79	−31.6	−45.44	−23.5

The compounds have excellent binding to both *E. coli* and *S. aureus* dihydrofolate reductase enzymes (PDB ID: 6XG5 and 6P9Z); good binding to the *S. aureus* DHPS enzyme (PDB ID: 1ad4); moderate to mild binding to muramyl ligase E of *E. coli* (PDB ID: 1E8C); and mild to poor binding to the *E. coli* DHPS (PDB ID: 5V7A), DNA gyrase B subunit (PDB ID: 4URO and 1KZN), and bacterial transpeptidase of *S. aureus* and *E. coli* (PDB ID: 5TW8 and 6NTW). Hence, it is possible that the compounds could destroy bacterial cells by interfering with multiple targets, including DHPS and DHFR enzymes.

## 4. Discussion

### 4.1. ADMET Analysis

We used the ADMET lab 2.0 platform to ascertain the compound′ physicochemical characteristics. The compounds showed a range of physiochemical and pharmacokinetic characteristics. Every compound was a HBD and acceptor. Every compound has rotatable bonds, with the exception of compound R1, which does not have. The compounds′ lower topological polar surface area (TPSA) values indicated that they were either less flexible than ampicillin or the same. LogS of most of the compounds was lower than −4 logmol/L except compound R1 which was −2.443 whereas ampicillin gave a log S value of −1.564 logmol/L. Based on their TPSA values, which were ≤ 56 Å, all of the compounds showed good oral bioavailability and high gastrointestinal absorption. This suggests that all of the compounds have good transport and cell permeability characteristics. The pharmacokinetic study showed that the compounds were not P‐gp (p‐glycoprotein) substrates because P‐gp will not pump them out of the cell, indicating that they are probably highly bioavailable. Blood brain barrier (BBB) permeation property was observed for all the compounds except compound R6, which was absent in ampicillin suggesting that compound R6 would have a greater propensity to cross the BBB. To prove that the drug‐likeness attribute complies with Lipinski′s RO5. The physicochemical characteristics of the compounds under test are listed in Table 2. All the compounds were found to have MW between the suggested range of 205–380. All the compounds had lipophilicity (logP) values that fell between the permissible range of 2.3–5.1. The majority of molecules with good drug‐likeness, according to the Lipinski “Rule of Five,” have logP less than or equal to 5, MW less than or equal to 500, the number of HB donors less than or equal to 5, and the number of HB acceptors less than or equal to 10. At least three of the four requirements must be met for a compound to be considered to follow Lipinski′s “Rule of Five.” It was discovered that every molecule complied fully with Lipinski′s RO5 [60]. The compounds were found to be excellent hERG blockers. The ADMET properties enable researchers to make the necessary changes to optimize the activity. All of the compounds examined demonstrated excellent expected human intestinal absorption. The compounds were discovered to have excellent human oral bioavailability (20% and 30%). The logS, predicted human oral absorption, and compliance with Jorgensen′s “Rule of Three” (logS> −5.7, Caco2>22 nm/s, and # primary metabolites < 7) [61], were useful in determining the oral availability of compounds. The nonactive transport for the gut–blood barrier was assessed from Caco‐2 cell permeability; all the 10 compounds had a Caco‐2 cell permeability value greater than −4.6.

There were no deviations from Jorgensen′s rule of three among the compounds. Medication binding to plasma proteins decreased the amount of medication that entered the bloodstream and therefore affected drug efficiency. Human serum albumin binding serves as a proxy for plasma–protein binding. All of the compounds were found to be in the range of 85.2%–98.8%, with ampicillin having a plasma–protein binding of 48.54%. As a result, the compounds are less likely to enter the bloodstream readily, making them less available to the target site.

### 4.2. Antimicrobial Activity: MIC Determination

Resistance to most drugs especially antimicrobials has been a major public health threat that seems to hinder the world′s achievement of the sustainable development goal (SDG) 3 aiming at good health and wellbeing for all by the year 2030 [62], for this reason, many scientists recently have resorted to the search for new antimicrobial agents that could help fight this public health menace. One of these efforts include the syntheses of some heterocyclic compounds like new benzodiazepines and benzothiazepines, which have been severally reported to possess many biological activities including antimicrobial potency [63]. Interestingly, thiazepines as one of these classes of compounds possess a broad spectrum of biological activities especially antimicrobial activities [64]. As a result, this study investigated the antimicrobial potential of some new 2,3‐dihydrobenzo[b][1,4]thiazepine derivatives that could be considered as medicine and food preservatives [65]. In this study, results from Tables 11, 12, 13, and 14 and Figure S41–S50 on the inhibitory activities of the test 2,3‐dihydrobenzo[b][1,4]thiazepine derivatives proved that they are potential antimicrobial agents with their MIC values ranging from 1.875–15 mg/mL. The in vitro antibacterial activity of the 10 synthetic compounds (R1–R10) was evaluated against four clinically relevant bacterial strains: *E. coli* (gram‐negative), *E. faecalis* (gram‐positive), *K. pneumoniae* (gram‐negative), and *S. aureus* (gram‐positive). The choice of organism was based on docking results. The activity was assessed in terms of MIC and MBC, with the MBC/MIC ratio used to classify compounds as either bactericidal (MBC/MIC ≤ 4) or bacteriostatic (MBC/MIC > 4).

The results indicate that the tested compounds exhibit a broad‐spectrum but variable antimicrobial activity. No single compound was generally effective against all four pathogens at low concentrations, highlighting organism‐ and compound‐specific interactions.

*E. coli* demonstrated the highest level of intrinsic resistance or tolerance to this series. Only R1 (MIC 7.5 mg/mL) and R6 (MIC/MBC 15 mg/mL) demonstrated this. For all other compounds (R2, R3, R4, R5, R7, R8, R9, and R10), no inhibitory activity was detected up to the tested concentrations (denoted as “Null”), suggesting that *E. coli* possesses effective efflux pumps, impermeable outer membrane barriers (particularly relevant for gram‐negatives), or intrinsic enzymatic degradation mechanisms against most of these chemical scaffolds.

*K. pneumoniae, S. aureus, and E. faecalis* were generally more susceptible. Several compounds showed promising activity against these organisms with MIC values as low as 1.875 mg/mL (e.g., R4 and R6 against *E. faecalis* and *S. aureus*). Notably, R3 exhibited consistent and relatively potent activity against *E. faecalis*, *K. pneumoniae*, and *S. aureus* with a low MIC of 3.75 mg/mL. Similarly, R6 showed good, broad activity against these three organisms with MICs ranging from 1.875 to 7.5 mg/mL. Compounds R6 and R7 also demonstrated consistent activity against most strains except *E. coli*, with MIC values ranging from 1.875 to 15 mg/mL.

### 4.3. Bactericidal Versus Bacteriostatic Profile

The MBC/MIC ratios indicate that most active compounds exhibit bactericidal activity (MBC/MIC ≤ 4). For example: R1 against *E. feacalis*, *K. pneumoniae*, and *S. aureus* (MBC/MIC = 1), R3 against *K. pneumoniae* and *S. aureus* (MBC/MIC = 4), R6 against *E. coli* and *K. pneumoniae* (MBC/MIC = 1), entries with MBC reported as null despite detectable MIC values (e.g., R1 against *E. coli*, R4 against *E. feacalis*, R5 against *K. pneumoniae*). This indicates a bacteriostatic effect at the tested concentrations, where the compound inhibits growth but does not kill the bacteria.

### 4.4. Structure Activity Relationship (SAR)

The inactivity of R2 (the 3‐nitro derivative) against all the test organisms suggests that the presence of polar groups at the meta position of phenyl group is not required for activity, suggesting the lack of polar groups at the active site of the molecule. The activity of R3 (the 4‐chloro derivative) against *E. feacalis*, *K. pneumonae* and *S. aureus* suggested that the presence of a polar group at the para position on the phenyl ring leads to a higher activity. R4 (the 3‐methylderivative exhibit variable activity, the low activity against *E. feacalis* suggests that the presence of no‐polar groups at the meta position is helpful for activity in *E. feacalis*, *K. pneumonae* and *S. aureus*. In R5 (the 4‐methyl derivative) has a reduced activity for *E. feacalis*, *K. pneumonae* and *S. aureus* compared with the 3‐methyl indicating that the methyl group has a better orientation for activity at the meta position than the para position. The presence of the *N,N*‐diethylamino group (R6) at the para position on the phenyl group allows the molecule to adopt the orientation necessary to elicit moderate activity against *E. coli* and *K. pneumonae* while showing very strong activity against *E. feacalis* and *S. aureus.* The 2‐hydroxy derivative (R7) and 2‐chloroderivative (R8) were found to be moderately active against *E. feacalis*, *K. pneumonae* and *S. aureus* but showed no activity against *E coli* at the test concentration suggesting that substituents at the ortho position are less active against the test organisms. The 3‐chloroderivative (R9) was moderately active against the test organisms, which confirms that the presence of polar groups at the meta position of the phenyl group does not lead to a higher activity among these compounds. In all, most of the activities of these compounds obtained were bactericidal with some few ones being bacteriostatic as shown in Tables 11, 12, 13, and 14. Most of these activities from the new 2,3‐dihydrobenzo[b][1,4]thiazepine derivatives can be attributed to the presence of electron withdrawing or electron donating substituents/groups on their aryl ring increasing their activities against most microbes as reported [66, 67]. Again, these inhibitory activities recorded falls in line with the results recorded against similar organisms like *E. coli* and *K. pneumonia* proving their potencies [68–70]. Therefore, these could be considered as promising drug candidates to help unearth and develop new drugs against microbial organisms that have defied conventional therapy antimicrobials. Though polymorphism is possible in the compounds in the active site slight structural variation is unlikely to heavily affect the activity of these compounds.

### 4.5. Mode of Action

The compounds are suggested to act by breaching the outer membrane due to the fact that the presence of alkyl groups as substituents leads to a higher activity. The alkyl group helps in crossing the outer membrane, making it easier to reach the active site for activity. This is also seen in the fact that the presence of polar groups tends to reduce the activity of the compounds, possibly due to extensive interaction of these polar molecules with the *N*‐acetylglucosamine (NAG) and *N*‐acetylmuramic acid (NAM) on the bacterial wall, limiting their ability to get to the active site for activity.

## 5. Conclusions

The 2,3‐dihydrobenzo[b][1,4]thiazepine derivatives were evaluated for their antimicrobial activity using agar well diffusion, microdilution, and biofilm inhibition assays. The results showed most of the compounds were active against *E. coli*, *K. pneumoniae*, *E. faecalis*, and *S. typhi* with MICs ranging from 1.88–15 mg/mL. In prediction of the mechanism of action, the computational molecular docking demonstrated that these heterocyclic compounds bind in a good to excellent fashion to five crucial target proteins from Gram (+ve) *S. aureus* and Gram (‐ve) *E. coli*, which include transpeptidase (PDB ID: 5TW8 and 6NTW), DNA gyrase B subunit (PDB ID: 4URN and 1KZN), dihydrofolate reductase (PDB ID: 6XG5 and 6P9Z), DHPS (PDB ID: 1 AD4 and 5V7A), and muramyl ligase (PDB ID: 4C13 and 1E8C). The ADMET study yielded varying results, indicating that some of the compounds could be promising lead molecules for the development of novel medications. Future work could include modification of the substituted to make them bulky so that they can interact with more groups at the active site to elicit stronger activity. In vivo studies using a rat model or Zebra fish model could provide useful information on these compounds or their derivatives; experimental ADMET studies could be used to confirm the in silico results.

## Author Contributions

**Felix Odame:** conceptualization of project, synthesis and characterization of compounds (excluding NMR), writing of first draft, editing of final draft. **Reuben Ayivor-Djanie:** discussion of antimicrobial work. **Tatenda Madanhire:** carrying out the NMR, review of manuscript. **Jaclyn Mann:** discussion of antimicrobial work, review of manuscript. **David Neglo:** discussion of antimicrobial work, review of manuscript. **Salifu Nanga:** carrying out statistical analysis on all initial data, review of manuscript. **Emmanuel Hamenoo:** Carrying out antimicrobial work, review of manuscript. **Cedric Amengor:** review of synthesis and characterization of compounds, review of manuscript. **Prince Biniyam:** carrying out the docking and its discussion. **Susanna Acheampong:** assisting with antimicrobial work. **Takalani Mulaudzi:** discussion of antimicrobial work.

## Funding

No funding was received for this project.

## Conflicts of Interest

The authors declare no conflicts of interest.

## Supporting information


**Supporting Information 1.** Additional supporting information can be found online in the Supporting Information section.

## Data Availability

The data that support the findings of this study are available from the corresponding author upon reasonable request.
