# Supplementary material for: Synthesis, Characterization, Antimicrobial Activity, Docking, and In Silico ADMET Analysis of Some New 2,3‐Dihydrobenzo[b][1,4]thiazepine Derivatives
Source: Biomed Res Int. 2026 May 30;2026:5076755. doi: 10.1155/bmri/5076755 (PMC13239082; doi:10.1155/bmri/5076755)
Supplement: Supplementary file 1 — Supporting Information 1. Additional supporting information can be found online in the Supporting Information section. [file BMRI-2026-5076755-s001.docx]

**Supporting Information**

**Table of content**

**1. Figures**

| **Fig. S1** | IR spectrum of 2,2,4-trimethyl-2,3-dihydrobenzo[b][1,4]thiazepine (**R1**). |
| --- | --- |
| **Fig. S2** | ^1^H NMR spectrum of 2,2,4-trimethyl-2,3-dihydrobenzo[b][1,4]thiazepine **(R1)** |
| **Fig. S3** | ^13^C NMR spectrum of 2,2,4-trimethyl-2,3-dihydrobenzo[b][1,4]thiazepine **(R1)** |
| **Fig. S4** | DEPT spectrum of 2,2,4-trimethyl-2,3-dihydrobenzo[b][1,4]thiazepine (**R1**) |
| **Fig. S5** | IR spectrum of (E)-2,2-dimethyl-4-(3-nitrostyryl)-2,3-dihydrobenzo[b][1,4] thiazepine (**R2**) |
| **Fig. S6** | ^1^H NMR spectrum of (*E*)-2,2-dimethyl-4-(3-nitrostyryl)-2,3-dihydrobenzo [b][1,4] thiazepine (**R2**) |
| **Fig. S7** | ^13^C NMR spectrum of (E)-2,2-dimethyl-4-(3-nitrostyryl)-2,3-dihydrobenzo [b][1,4] thiazepine (**R2**) |
| **Fig. S8** | DEPT spectrum of (*E*)-2,2-dimethyl-4-(3-nitrostyryl)-2,3-dihydrobenzo[b][1,4] thiazepine (**R2**) |
| **Fig. S9** | IR spectrum of (*E*)-4-(4-chlorostyryl)-2,2-dimethyl-2,3-dihydrobenzo[b][1,4] thiazepine (**R3**) |
| **Fig. S10** | ^1^H NMR spectrum of (*E*)-4-(4-chlorostyryl)-2,2-dimethyl-2,3-dihydrobenzo [b][1,4]thiazepine (**R3**) |
| **Fig. S11** | ^13^C NMR spectrum of (*E*)-4-(4-chlorostyryl)-2,2-dimethyl-2,3-dihydrobenzo [b][1,4]thiazepine (**R3**) |
| **Fig. S12** | DEPT spectrum of (*E*)-4-(4-chlorostyryl)-2,2-dimethyl-2,3-dihydrobenzo [b][1,4] thiazepine (**R3**) |
| **Fig. S13** | IR spectrum of (*E*)-2,2-dimethyl-4-(3-methylstyryl)-2,3-dihydrobenzo[b][1,4] thiazepine (**R4**) |
| **Fig. S14** | ^1^H NMR spectrum of (*E*)-2,2-dimethyl-4-(3-methylstyryl)-2,3-dihydrobenzo [b][1,4]thiazepine (**R4**) |
| **Fig. S15** | ^13^C NMR spectrum of (*E*)-2,2-dimethyl-4-(3-methylstyryl)-2,3-dihydrobenzo [b][1,4]thiazepine (R4) |
| **Fig. S16** | DEPT spectrum of (*E*)-2,2-dimethyl-4-(3-methylstyryl)-2,3-dihydrobenzo [b][1,4] thiazepine (**R4**) |
| **Fig. S17** | IR spectrum of (E)-2,2-dimethyl-4-(4-methylstyryl)-2,3-dihydrobenzo [b][1,4]thiazepine (**R5**) |
| **Fig. S18** | 1H NMR spectrum of (*E*)-2,2-dimethyl-4-(4-methylstyryl)-2,3-dihydrobenzo [b][1,4]thiazepine  **(R5)** |
| **Fig. S19** | ^13^C NMR spectrum of (*E*)-2,2-dimethyl-4-(4-methylstyryl)-2,3-dihydrobenzo [b][1,4]thiazepine (**R5**) |
| **Fig. S20** | DEPT spectrum of (*E*)-2,2-dimethyl-4-(4-methylstyryl)-2,3-dihydrobenzo[b] [1,4]thiazepine **(R5)** |
| **Fig. S21** | IR spectrum of (*E*)-4-(2-(2,2-dimethyl-2,3-dihydrobenzo[b][1,4]thiazepin-4-yl)vinyl)-N,N-diethylaniline (**R6**) |
| **Fig. S22** | ^1^H NMR spectrum of (*E*)-4-(2-(2,2-dimethyl-2,3-dihydrobenzo [b][1,4] thiazepin-4-yl)vinyl)-N,N-diethylaniline (**R6**) |
| **Fig. S23** | ^13^C NMR spectrum of (*E*)-4-(2-(2,2-dimethyl-2,3-dihydrobenzo [b][1,4] thiazepin-4-yl)vinyl)-N,N-diethylaniline (**R6**) |
| **Fig. S24** | DEPT spectrum of (*E*)-4-(2-(2,2-dimethyl-2,3-dihydrobenzo[b][1,4]thiazepin-4-yl)vinyl)-N,N-diethylaniline (**R6**) |
| **Fig. S25** | IR spectrum of (*E*)-2-(2-(2,2-dimethyl-2,3-dihydrobenzo[b][1,4]thiazepin-4-yl)vinyl)phenol (**R7**) |
| **Fig. S26** | ^1^H NMR spectrum of (*E*)-2-(2-(2,2-dimethyl-2,3-dihydrobenzo[b][1,4] thiazepin-4-yl)vinyl)phenol (**R7**) |
| **Fig. S27** | ^13^C NMR spectrum of (*E*)-2-(2-(2,2-dimethyl-2,3-dihydrobenzo[b][1,4] thiazepin-4-yl)vinyl)phenol (**R7**) |
| **Fig. S28** | DEPT spectrum of (*E*)-2-(2-(2,2-dimethyl-2,3-dihydrobenzo[b][1,4]thiazepin-4-yl)vinyl)phenol (**R7**) |
| **Fig. S29** | IR spectrum of (*E*)-4-(2-chlorostyryl)-2,2-dimethyl-2,3-dihydrobenzo [b][1,4]thiazepine (**R8**) |
| **Fig. S30** | ^1^H NMR spectrum of (*E*)-4-(2-chlorostyryl)-2,2-dimethyl-2,3-dihydrobenzo [b][1,4]thiazepine (**R8**) |
| **Fig. S31** | ^13^C NMR spectrum of (*E*)-4-(2-chlorostyryl)-2,2-dimethyl-2,3-dihydrobenzo [b][1,4]thiazepine (**R8**) |
| **Fig. S32** | DEPT spectrum of (*E*)-4-(2-chlorostyryl)-2,2-dimethyl-2,3-dihydrobenzo [b][1,4]thiazepine (**R8**) |
| **Fig. S33** | IR spectrum of (*E*)-4-(3-chlorostyryl)-2,2-dimethyl-2,3-dihydrobenzo[b][1,4] thiazepine (**R9**) |
| **Fig. S34** | ^1^H NMR spectrum of (*E*)-4-(3-chlorostyryl)-2,2-dimethyl-2,3-dihydrobenzo [b][1,4]thiazepine (**R9**) |
| **Fig. S35** | ^13^C NMR spectrum of (E)-4-(3-chlorostyryl)-2,2-dimethyl-2,3-dihydrobenzo [b][1,4]thiazepine (**R9**) |
| **Fig. S36** | DEPT spectrum of (*E*)-4-(3-chlorostyryl)-2,2-dimethyl-2,3-dihydrobenzo[b][1,4] thiazepine **(R9)** |
| **Fig. S37** | IR spectrum of (*E*)-2,2-dimethyl-4-styryl-2,3-dihydrobenzo[b][1,4]thiazepine (**R10)** |
| **Fig. S38** | ^1^H NMR spectrum of (*E*)-2,2-dimethyl-4-styryl-2,3-dihydrobenzo[b][1,4] thiazepine (**R10**) |
| **Fig. S39** | ^13^C NMR spectrum of (*E*)-2,2-dimethyl-4-styryl-2,3-dihydrobenzo[b][1,4] thiazepine (**R10**) |
| **Fig. S40** | DEPT spectrum of (*E*)-2,2-dimethyl-4-styryl-2,3-dihydrobenzo [b][1,4] thiazepine (R**10**) |
| **Fig. S41** | A diagram of MIC Setup for Compound **R1.** |
| **Fig. S42** | A diagram of MIC Setup for Compound **R2** |
| **Fig. S43** | A diagram of MIC Setup for compound **R3** |
| **Fig. S44** | A diagram of MIC Setup for Compound **R4** |
| **Fig. S45** | A diagram of MIC Setup for Compound **R5** |
| **Fig. S46** | A diagram of MIC Setup for Compound **R6** |
| **Fig. S47** | A diagram of MIC Setup for Compound **R7** |
| **Fig. S48** | A diagram of MIC Setup for Compound **R8** |
| **Fig. S49** | A diagram of MIC Setup for Compound **R9** |
| **Fig. S50** | A diagram of MIC Setup for Compound **R10** |

**2. Tables**

| **Table S1** | Spectral IR band frequencies (cm^-1^) of compounds **R1**–**R10**. |
| --- | --- |
| **Table S2** | Results of Antimicrobial work |
